# Comparison of shoulder kinetics between running overhead throws with circular and whip-like wind up in elite handball players

**DOI:** 10.3389/fspor.2026.1820988

**Published:** 2026-06-19

**Authors:** Roland van den Tillaar

**Affiliations:** Department of Sports Science and Physical Education, Nord University, Levanger, Norway

**Keywords:** ball velocity, distraction, forces, internal shoulder rotation, torque

## Abstract

**Introduction:**

The aim of the study was to compare shoulder kinetics between the whip-like and circular wind-ups in running throws in handball.

**Methods:**

Twenty-five elite handball players performed running throws with a circular and whip-like wind up while a markerless motion capture system consisting of eight cameras sampling at 250 Hz measured and calculated ball velocity, joint angle, angular velocity, shoulder torques and force during the throws.

**Results:**

The main findings were that peak ball and maximal angular external shoulder velocity were higher with the circular wind up together with time of occurrence to closer ball release of peak external rotation torque and peak posterior shear forces compared with the whip-like wind up, while a larger external shoulder rotation angle with a earlier time of occurrences was observed with the whip-like wind up.

**Discussion:**

It was concluded that although differences in shoulder kinematics between the two wind-ups were observed it did not result in differences in peak shoulder torque and forces, only in timing. Thereby, both throws seem to cause similar stress upon the shoulder. Thereby, both throws seem to cause similar stress upon the shoulder. While no statistically significant differences in peak shoulder torques and forces were observed between techniques; however, due to the sample size, variability in throwing experience, and the absence of equivalence testing, these findings should not be interpreted as evidence that both techniques impose equivalent shoulder stress.

## 1 Introduction

Handball is a high-intensity sport characterized by dynamic movements, frequent overhead throws, and rapid changes in direction, all performed with the aim of scoring goals. The throwing actions, while essential to gameplay, place substantial stress on the shoulder joint, making it particularly susceptible to injury. Shoulder injuries are common in handball, with a reported prevalence of 17%–41% among youth and adult players (1–3). Such injuries are often attributed to the repetitive loading placed on the shoulder during throwing, compounded by variations in technique and movement patterns among athletes.

In handball, two primary types of wind-ups are used during throwing: the whip-like and the circular wind-up. The whip-like wind-up involves a rapid, slingshot-like acceleration of the arm, whereas the circular wind-up consists of a more rotational, sweeping arm motion (4). These differing techniques may impose distinct mechanical demands on the shoulder joint, potentially influencing both the kinematics and kinetics of the throwing motion.

Previous studies have examined the biomechanics of handball throwing and highlighted the importance of shoulder movements for generating maximal ball velocity across different throwing techniques (4–6). van den Tillaar et al. (4) reported that, in standing throws, the circular wind-up produced higher ball velocities than the whip-like wind-up, whereas the whip-like wind-up resulted in a significantly shorter total throwing time, both characteristics offering potential advantages in competition. They also found that the whip-like wind-up produced greater external shoulder rotation during the cocking phase, which may increase anterior, medial, and compressive forces around the elbow and shoulder. When repeatedly performed, this increased loading could contribute to overuse injuries of the rotator cuff muscles and the ligaments responsible for decelerating external rotation.

Despite these insights, no detailed kinetic analysis of shoulder loading during handball throws (7) has been conducted to date. In contrast, several studies in baseball have investigated shoulder kinetics during pitching (8–10) and have suggested that specific throwing mechanics may elevate injury risk (11, 12). For example, Manzi et al. (13) reported that increased shoulder abduction at ball release and greater maximal external rotation were associated with higher superior and distraction forces at the shoulder. Werner et al. (14) found that pitchers with more limited external rotation at the end of the cocking phase, and with lower external rotation and abduction torques, experienced reduced shoulder distraction forces.

However, these findings stem from baseball pitching, which typically involves only a single forward step and therefore differs substantially from the running throw used in handball. In javelin throwing—more biomechanically similar to the handball running throw, only one study has reported shoulder kinetics (15). They found that energy flow during the acceleration phase and resultant joint torques during the deceleration phase are linked, and that these demands can be modified by changes in joint angles at release.

To date, no detailed kinetic analysis of the shoulder joint in handball has been performed that, together with kinematic data, could provide deeper insight into shoulder loading during the two primary wind-up techniques. Such knowledge is essential for both injury prevention and performance enhancement. Therefore, the present study aims to address this gap by comparing shoulder kinematics and kinetics between the whip-like and circular wind-ups during running throws in handball. By examining the biomechanical differences between these techniques, this study seeks to provide valuable insights into how each wind-up contributes to shoulder loading and how adjustments in technique may help reduce injury risk in handball athletes. It was hypothesized that higher peak torques and forces would occur during standing circular wind-up throws as van den Tillaar et al. (4) reported that these throws resulted in higher ball velocities and thereby potentially also result in higher shoulder peak torques and forces than whip-like wind-up throws.

## 2 Materials and methods

### 2.1 Participants

Twenty-five elite male handball players (age 18.8 ± 3.3 years, body mass 79.0 ± 7.4 kg, body height 1.84 ± 0.06 m) competing in the highest national division volunteered to participate in this study. Players represented all field positions except goalkeeper. Written and oral informed consent was obtained from all players, and from legal guardians for those under 18 years of age. The experimental protocol was approved by the Norwegian Ethics Committee, REK Midt (ref. 7,189), and adhered to the Declaration of Helsinki.

### 2.2 Procedure

After a general individual warm-up of 15 min, including passes and throws to properly warm up the shoulder, players were instructed to perform maximal-effort throws using a standard handball (0.46 kg). Each throw consisted of three preliminary steps (running throw) followed by either a circular wind-up or a whip-like wind-up (4), directed straight toward the handball goal. The order of the throws was counter balanced randomized to avoid an influence of testing order. These throwing techniques were selected because they typically produce the highest ball velocities among handball throws (6), and therefore likely generate the greatest shoulder torques and forces. Each player performed three throws with each wind-up technique. Kinematics and kinetics of the players and the ball were recorded using markerless motion capture (Simi Nemo; Simi Reality Motion Systems GmbH, Unterschleissheim, Germany) with eight cameras sampling at 250 Hz. Each camera captured the subject and the ball simultaneously. The software separates the subject and the ball from the background to create a 2D silhouette. Advanced AI learning algorithms, combined with computer vision techniques, track body landmarks and movement frames by frame. Then the system combines data from all camera angles to compute the 3D silhouette for the subject and the ball separately. Thereby, compute a 3D skeletal structure, that maps to the anatomical structure of the athlete, defining the precise 3D locations of body joints. All calculated data-rows were 2nd order lowpass filtered with 20 Hz frequency. This system has shown that key biomechanical variables were accurate with mean relative bias and random error of 0.3% and 3.8%, respectively (16). When the ball distance separated the hand very rapidly this was identified as ball release and ball release velocity was automatically calculated (17). The fastest throw for each wind-up was selected for further analysis, as this was expected to produce the highest shoulder joint loading.

Only the phase from the onset of forward ball acceleration to 0.05 s after ball release (deceleration phase) was analyzed, as this interval contains the highest angular joint velocities and joint kinetics, and therefore likely the greatest shoulder load (4, 5). Shoulder abduction, horizontal extension/flexion, and internal rotation angles (Euler angles in XZY order) at ball release (defined as the moment when the distance between the ball and wrist increases rapidly), as well as maximal external rotation, were extracted together with their peak angular velocities and the timing of these events, calculated by the Simi motion software. Timing was expressed relative to ball release.

**Figure 1:**
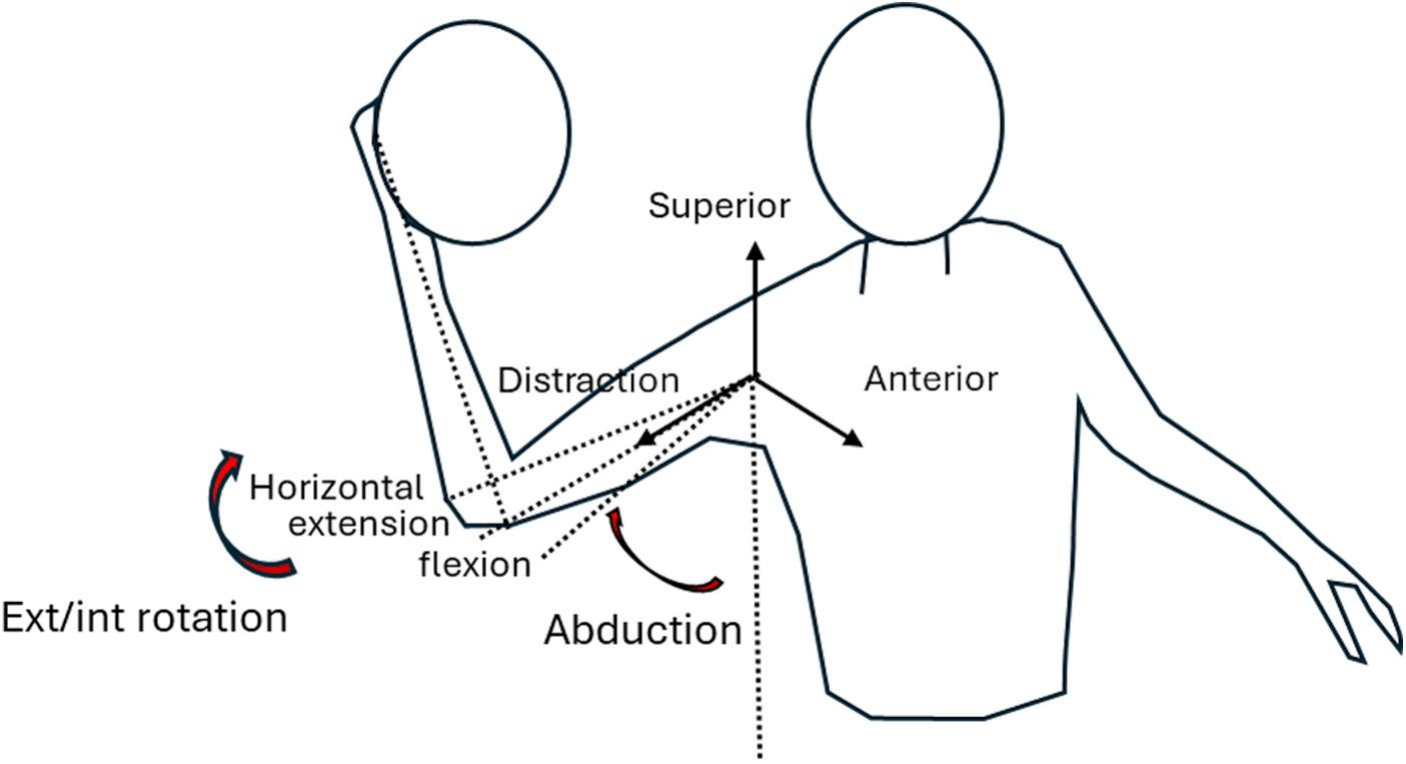
Definition of angles, torques and forces around the shoulder.

**Figure 2:**
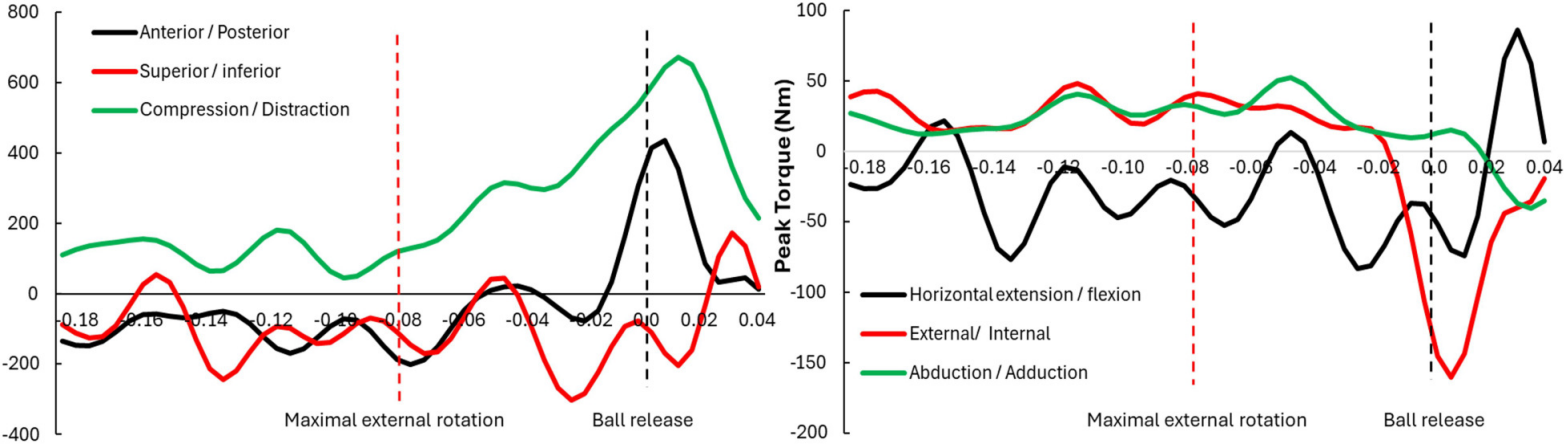
A typical example of development of the torques and forces in the three different directions around the shoulder.

Furthermore, maximal anterior/posterior, superior/inferior, and distraction forces acting on the shoulder joint were calculated using inverse dynamics based on ball acceleration and the hand, forearm, and upper-arm segments identified by the system. Shoulder abduction, horizontal extension/flexion, and external/internal rotation torques during the analyzed phase were also computed by the software (Figure 1, 2).

### 2.3 Statistical analysis

All statistical analyses were performed using JASP (version 0.95.4, University of Amsterdam, Amsterdam, The Netherlands). Normality was verified using the Shapiro–Wilk test. To compare kinematics and kinetics parameters between the two wind-up throws, a paired sampled *t*-test was used. The level of significance was set at *p* < 0.05, and all data are expressed as mean ± standard deviation (SD). The effect size (ES) was evaluated with Cohen's d where <0.20 constitutes a small effect, Cohen's d ≤ 0.20 < 0.80 constitutes a medium effect, and ≥0.80 Cohen's d constitutes a large effect (18). All analyses were carried out using JASP software (version 0.95.1, University of Amsterdam, Amsterdam, The Netherlands), with significance level set at *p* < 0.05.

## 3 Results

Significantly higher ball release velocities were achieved with the circular wind-up compared with the whip-like wind-up (25.5 ± 2.3 vs. 24.7 ± 2.3 m/s; Table 1). The whip-like wind-up showed a significantly earlier onset of ball acceleration than the circular wind-up (Table 1). No significant differences in joint angles at ball release were found between the two wind-ups. However, maximal external shoulder rotation angle was significantly greater in the whip-like wind-up, and it occurred earlier relative to ball release compared with the circular wind-up (Table 1). Only the peak angular velocity of external shoulder rotation differed significantly between techniques, with higher values observed in the circular wind-up. No other significant differences were found in the remaining peak angular velocities or in the timing of their occurrence (Table 1).

**Table 1:** Main ± SD shoulder torques and forces and their timing during whip-like and circular wind up of running throw. .

	Whip-like	Circular	p	Cohen's d
Forces (*N*)				
Posterior	348.3 ± 157.5	324.3 ± 156.0	0.492	0.14
Superior	256.1 ± 140.3	221.2 ± 121.1	0.249	0.24
Distraction	794.9 ± 179.5	845.2 ± 172.6	0.106	0.34
Anterior	−357.1 ± 134.2	−302.9 ± 99.1	0.083	−0.36
Inferior	−404.5 ± 176.7	−395.9 ± 165.2	0.84	−0.04
Torques (Nm)				
Horizontal extension	72.8 ± 47.7	65.6 ± 38.3	0.519	0.13
External rotation	89.2 ± 31.1	83.8 ± 27.6	0.468	0.15
Adduction	71.6 ± 13.5	70.3 ± 14.8	0.18	−0.28
Horizontal flexion	−126.7 ± 58.3	−120.9 ± 54.1	0.65	−0.09
Internal rotation	−142.1 ± 63.2	−133.2 ± 70.7	0.473	−0.15
Abduction	−38.6 ± 12.2	−35.8 ± 12.3	0.101	−0.34
Timing torques and force (s)				
Posterior	0.005 ± 0.014	−0.004 ± 0.017	0.022*	0.50
Superior	−0.074 ± 0.032	−0.077 ± 0.026	0.647	0.09
Distraction	0.000 ± 0.008	0.000 ± 0.007	0.819	0.05
Anterior	−0.090 ± 0.032	−0.081 ± 0.020	0.331	0.20
Inferior	−0.006 ± 0.024	−0.006 ± 0.020	1.000	0.01
Superior-inferior	−0.093 ± 0.008	−0.077 ± 0.026	0.343	0.20
Distraction	0.026 ± 0.016	0.000 ± 0.007	0.296	0.31
Horizontal extension	−0.068 ± 0.039	−0.078 ± 0.050	0.415	0.17
External rotation	−0.088 ± 0.034	−0.065 ± 0.008	0.028*	0.47
Adduction	−0.054 ± 0.020	−0.047 ± 0.016	0.587	0.11
Horizontal flexion	0.004 ± 0.016	0.001 ± 0.019	0.627	0.10
Internal rotation	−0.007 ± 0.010	0.003 ± 0.019	0.23	0.25
Abduction	−0.034 ± 0.009	−0.032 ± 0.011	0.498	0.14

**Figure 3:**
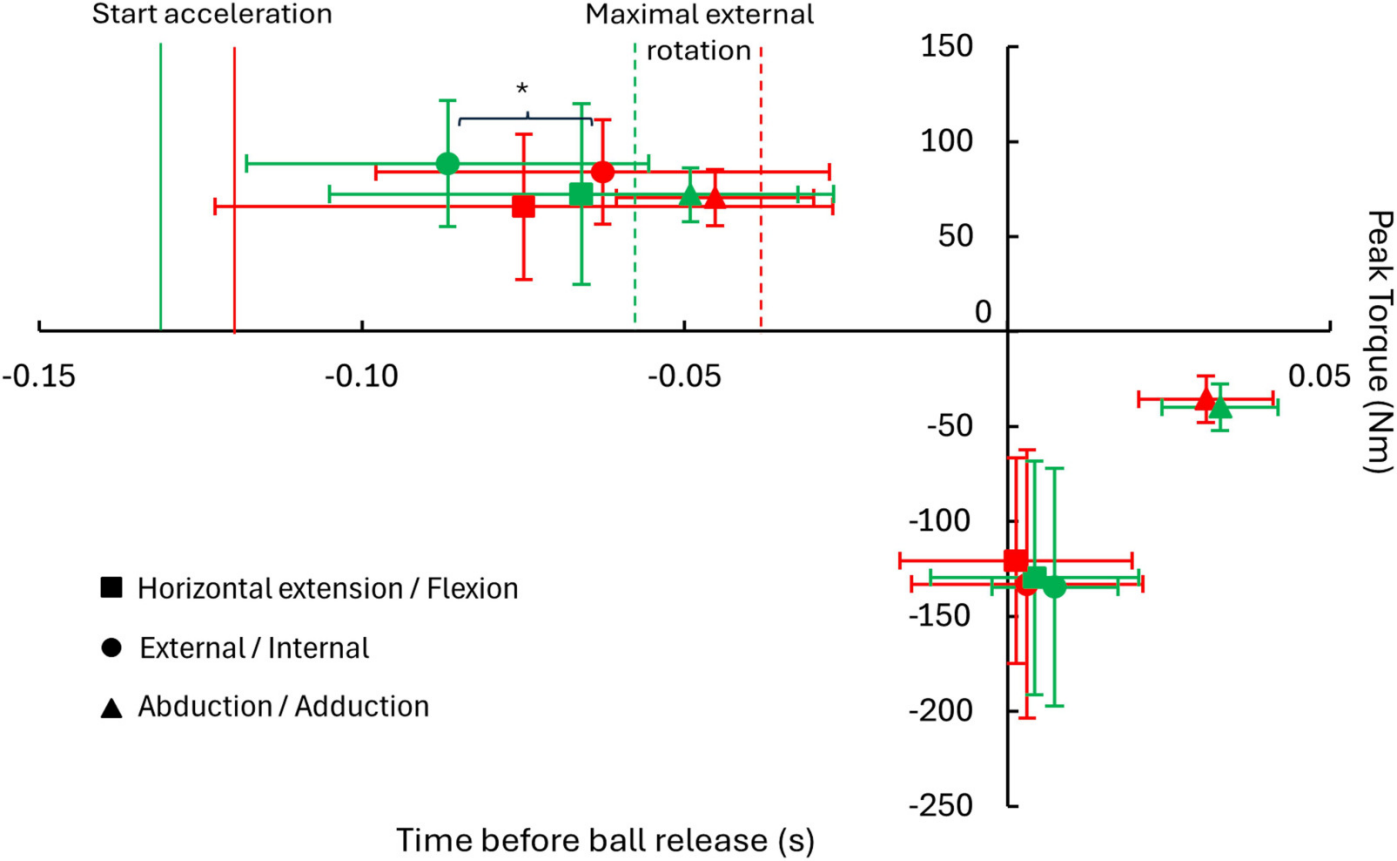
Average peak ± SD shoulder torques during whip-like (green) and circular wind up (red) of running throw with mean start ball acceleration and maximal external shoulder rotation during both wind ups. * indicates a significant difference in time of occurrence between the two wind ups on a *p* < 0.05 level.

**Figure 4:**
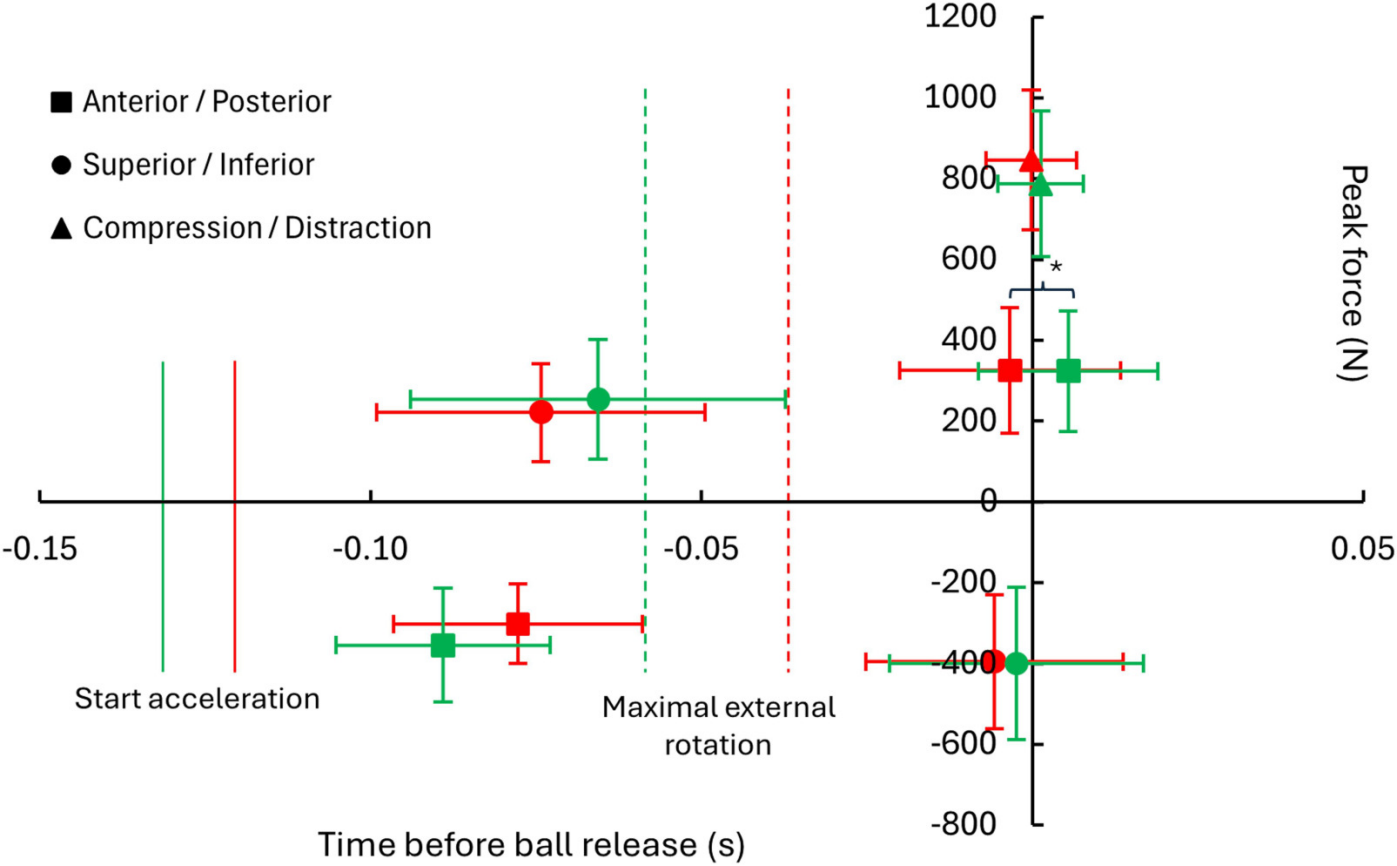
Average peak ± SD shoulder joint forces during whip-like (green) and circular wind up (red) of running throw with mean start ball acceleration and maximal external shoulder rotation during both wind ups. * indicates a significant difference in time of occurrence between the two wind ups on a *p* < 0.05 level.

No significant differences in shoulder joint torques were found in any direction, nor were any differences observed in peak forces between the two wind-ups (*p* ≥ 0.101, Cohen's d ≤ 0.34). However, the circular wind-up showed a significantly later occurrence, closer to ball release, of peak external rotation torque (*p* = 0.028, Cohen's d = 0.47) and peak posterior shear force (*p* = 0.022, Cohen's d = 0.50) compared with the whip-like wind-up (Figures 3, 4).

## 4 Discussion

The purpose of the current study was to compare shoulder kinematics and kinetics between the whip-like and circular wind-ups in running throws in handball. The main findings were that peak ball and maximal angular external shoulder velocity were higher with the circular wind up together with time of occurrence to closer ball release of peak external rotation torque and peak posterior shear force compared with the whip-like wind up, while a larger external shoulder rotation angle with a earlier time of occurrences was observed with the whip-like wind up.

The reported ball velocities in our study were comparable to an earlier study on running throws (6), while velocities were a bit higher with a later start of ball acceleration forwards (Table 1) than van den Tillaar et al. (4), who compared the different wind-ups, but from a standing throw condition. Due to the three preliminary steps, ball velocities were higher as the ball does not have to start from zero during the ball acceleration phase. Nonetheless, the differences in ball velocity and timing between the two wind ups were similar in both studies. During the cocking phase, larger maximal external rotation angles were observed than in standing throws (4), which are probably the result of the moment of inertia of the ball when the ball moves backwards, while the player is running forward, thereby preloading the shoulder muscles for the stretch shortening movement. During the whip-like wind up the ball moves straight up and backwards, which causes a higher peak external shoulder angle longer before ball release compared with the circular wind up.

Although the maximal angular external rotation velocity was higher during the circular wind-up than during the whip-like wind-up, it caused a lower maximal external rotation angle (156 vs. 152˚), which is likely caused by the longer backward cocking movement during this wind up. While this maximal angular velocity was greater, it did not result in higher anterior or superior forces or torques. This is probably because the time interval from maximal external rotation angular velocity to the point of maximal external shoulder rotation was longer for the circular compared with the whip-like wind up (0.073 vs. 0.053 s, Table 1). These differences in timing between these two events probably also cause the significant difference in timing of external rotation torques (Figure 3) in this cocking phase.

Similar anterior (300 vs. 200–300 N) and superior (221 ± 121 vs. 200–300 N) forces were observed during the cocking phase and peak distraction (845 ± 150 vs. 900–1,100 N), posterior (324 ± 155 vs.∼400 N) and inferior forces (395 ± 165 vs. 300–500 N) around ball release as in baseball (8, 10, 13, 14) even with difference in ball weights between the two sports (0.46 vs. 0.145 kg). Due to the lower ball weights in baseball, higher peak internal rotation (6,500–7,000 vs. 2,828 ˚/s) and abduction velocity (300–500 vs. 240 ˚/s), while peak horizontal flexion velocity was higher in the present study (744 ± 244 vs. 400–600°/s) compared with the baseball studies (8, 11). This indicates that the difference in ball weight has an effect upon peak throwing kinematics (19), but that the peak forces around the shoulder remain pretty comparable. Due to the similar forces during the cocking phase, external rotation (83 ± 27 vs. ∼90 N m), horizontal flexion (65 ± 38 vs. 40–60 N·m) and abduction (70 ± 14 vs.64–80) torques were comparable with elite baseball players and javelin throwers (15). These high forces and torques in this phase are associated with high stress on the anterior capsule and labrum (20, 21).

However, during the acceleration and follow-through phases, peak internal rotation torque (120 ± 50 N m vs. 80–100 N m) and horizontal-flexion torque (120 ± 54 N m vs. 50–70 N m) were substantially higher than those reported in baseball pitching (13, 14, 22). The higher horizontal flexion torque in handball is likely explained by the greater peak horizontal-flexion velocity (Table 1), combined with the larger ball mass compared with baseball.

Even though peak internal rotation velocity in handball was approximately half that observed in baseball, maximal internal rotation torque was higher. This can be explained by the impulse–momentum relationship (∫F * t = m v). In both throwing types, the ball accelerates from zero velocity to peak velocity at release, but with different ball masses. Using the peak velocity reported by Werner et al. (14) for baseball (40 m/s with a 0.145-kg ball), the resulting impulse is 5.8 N s. In contrast, based on the present study's handball data (v = 25.5 m/s, m = 0.46 kg), the impulse is 11.73 N s. Because total throwing time was comparable between studies Werner et al. (14), the force at ball release must therefore be higher in handball. Furthermore, because the distance from the shoulder to the ball at release (radius) is similar between handball players in the present study and the baseball pitchers in Werner et al. (14) were of similar height (1.88 m), torque (*τ* = r * F) will also be higher in handball. These high peak internal rotation torques immediately after ball release (Figure 3), combined with the large distraction forces observed (Figure 4), present a substantial challenge during the deceleration and follow-through phases. These phases involve significant braking forces and require coordinated scapular and humeral control to maintain glenohumeral stability during rapid arm slowdown. The resulting eccentric loading of the rotator-cuff muscles and the large distraction forces pulling the humeral head away from the glenoid have been well documented as key stressors in overhead throwing (11).

The study has some limitations as we tested players from different positions and thereby had diverse experiences with the two wind up techniques. So do pivot players use mainly the whip-like wind up, while the back players use in general the circular wind up. This could have an influence upon kinematics and kinetics. Furthermore, the number of players was not so high to make sub analyses per position. In addition, only the fastest throw from each player was used for further analysis as we expected that with this throw the highest torques and peak forces would appear. However, it is possible that with throws with lower maximal ball velocities, even higher torques and peak forces occur due to different timing of the segments during the throws. This should be investigated in future studies. Another limitation is that there is much variability in throwing technique between players, which also causes challenges to compare the different events with each other and their kinetics. Finally, the present study tested shoulder kinetics between the two wind-ups one time with players that did not have shoulder pain. Therefore, it is difficult to state if the maximal torque and forces are too high for the players to develop shoulder injuries. Studies investigating players with and without shoulder pain or over several times during the season should be performed to get more insight over what maximal torques and forces would be harmful for players.

## 5 Conclusion and practical recommendations

It was concluded that although small differences in shoulder kinematics between the two wind-ups were observed it did not result in differences in peak shoulder torque and forces, only in timing. Thereby, both throws seem to cause similar stress upon the shoulder. While no statistically significant differences in peak shoulder torques and forces were observed between techniques; however, due to the sample size, variability in throwing experience, and the absence of equivalence testing, these findings should not be interpreted as evidence that both techniques impose equivalent shoulder stress. Future studies should include more players from same playing positions to explore throwing kinematics and kinetics between positions, which could give more information about the throwing shoulder load for playing positions specifically. Furthermore, studies examining variability within players and over a period of time should be conducted as these probably change when throwing performance changes due to training or injury.

## Data Availability

The raw data supporting the conclusions of this article will be made available by the authors, without undue reservation.

## References

[1] Moller M Attermann J Myklebust G Wedderkopp N. Injury risk in Danish youth and senior elite handball using a new SMS text messages approach. Br J Sports Med. (2012) 46:531–7. 10.1136/bjsports-2012-091022 22554848

[2] Aasheim C Stavenes H Andersson SH Engbretsen L Clarsen B. Prevalence and burden of overuse injuries in elite junior handball. BMJ Open Sport Exerc Med. (2018) 4:e000391. 10.1136/bmjsem-2018-000391 30018791 PMC6045727

[3] Asker M Brooke HL Waldén M Tranaeus U Johansson F Skillgate E. Risk factors for, and prevention of, shoulder injuries in overhead sports: a systematic review with best-evidence synthesis. Br J Sports Med. (2018) 52:1312–9. 10.1136/bjsports-2017-098254 29581141

[4] Van Den Tillaar R Zondag A Cabri J. Comparing performance and kinematics of throwing with a circular and whip-like wind up by experienced handball players. Scand J Med Sci Sports. (2013) 23:e373–380. 10.1111/sms.12091 23782364

[5] Wagner H Buchecker M Von Duvillard S Muller E. Kinematic comparison of team handball throwing with different arm positions. Int J Sports Physiol Perf. (2010) 5:469–83. 10.1123/ijspp.5.4.469 21266732

[6] Wagner H Pfusterschmidt J Von Duvillard SP Müller E. Performance and kinematics of various throwing techniques in team-handball. J Sports Sci Med. (2011) 10:73–80. 24149298 PMC3737895

[7] Skejø SD Møller M Bencke J Sørensen H. Shoulder kinematics and kinetics of team handball throwing: a scoping review. Hum Mov Sci. (2019) 64:203–12. 10.1016/j.humov.2019.02.006 30784891

[8] Fleisig GS Kingsley DS Loftice JW Dinnen KP Ranganathan R Dun S. Kinetic comparison among the fastball, curveball, change-up, and slider in collegiate baseball pitchers. Am J Sports Med. (2006) 34:423–30. 10.1177/0363546505280431 16260466

[9] Werner SL Guido JA Jr Stewart GW Mcneice RP Vandyke T Jones DG. Relationships between throwing mechanics and shoulder distraction in collegiate baseball pitchers. J Shoulder Elbow Surg. (2007) 16:37–42. 10.1016/j.jse.2006.05.007 17169584

[10] Urbin M Fleisig GS Abebe A Andrews JR. Associations between timing in the baseball pitch and shoulder kinetics, elbow kinetics, and ball speed. Am J Sports Med. (2013) 41:336–42. 10.1177/0363546512467952 23204507

[11] Fleisig GS Andrews JR Dillman CJ Escamilla RF. Kinetics of baseball pitching with implications about injury mechanisms. Am J Sports Med. (1995) 23:233–9. 10.1177/036354659502300218 7778711

[12] Chalmers PN Wimmer MA Verma NN Cole BJ Romeo AA Cvetanovich GL. The relationship between pitching mechanics and injury: a review of current concepts. Sports Health. (2017) 9:216–21. 10.1177/1941738116686545 28107113 PMC5435152

[13] Manzi JE Dowling B Trauger N Fu MC Hansen BR Dines JS. The influence of shoulder abduction and external rotation on throwing arm kinetics in professional baseball pitchers. Shoulder Elbow. (2022) 14:90–8. 10.1177/17585732211010300 35845618 PMC9284251

[14] Werner SL Gill TJ Murray TA Cook TD Hawkins RJ. Relationships between throwing mechanics and shoulder distraction in professional baseball pitchers. Am J Sports Med. (2001) 29:354–8. 10.1177/03635465010290031701 11394608

[15] Köhler H-P Schödlbauer M Witt M. How the acceleration phase influences energy flow and the resulting joint moments of the throwing shoulder in the deceleration phase of the javelin throw. Front Sports Act Living. (2024) 6:1445455. 10.3389/fspor.2024.1445455 39534526 PMC11556347

[16] Bissas A Cronin NJ. Evaluating markerless biomechanical analysis in a real-world pole vault competition setting. PLoS One. (2026) 21:e0329987. 10.1371/journal.pone.0329987 41894358 PMC13028377

[17] Van Den Tillaar R Ettema G. A three-dimensional analysis of overarm throwing in experienced handball players. J Appl Biomech. (2007) 23:12–9. 10.1123/jab.23.1.12 17585175

[18] Cohen J. Statistical Power Analysis for the Behavioral Sciences. Hillsdale, NJ, England: Lawrence Erlbaum Associates (1988).

[19] Van Den Tillaar R Ettema G. A force-velocity relationship and coordination patterns in overarm throwing. J Sports Sci Med. (2004) 3:211. 24624005 PMC3938059

[20] Zheng N Fleisig GS Andrews JR. Biomechanics and injuries of the shoulder during throwing. Athl Ther Today. (1999) 4:6. 10.1123/att.4.4.6

[21] Naito K Takagi T Kubota H Maruyama T. Relationship between shoulder forces, shoulder joint shear stress, and throwing velocity during baseball pitching. Proc Instit Mech Eng Part P J Sports Eng Technol. (2019) 233:489–502. 10.1177/1754337119852458

[22] Sabick MB Torry MR Kim Y-K Hawkins RJ. Humeral torque in professional baseball pitchers. Am J Sports Med. (2004) 32:892–8. 10.1177/0363546503259354 15150034

